# Protective effects of *Ginkgo Biloba* Dropping Pills against liver ischemia/reperfusion injury in mice

**DOI:** 10.1186/s13020-020-00404-z

**Published:** 2020-11-19

**Authors:** Zheng Wang, Ping Zhang, Qingqing Wang, Xueping Sheng, Jianbing Zhang, Xiaoyan Lu, Xiaohui Fan

**Affiliations:** 1 Pharmaceutical informatics institute, College of Pharmaceutical Science, Zhejiang University, 310058 Hangzhou, China; 2 State Key Laboratory of Modern Chinese Medicine, Tianjin University of Traditional Chinese Medicine, 301617 Tianjin, China; 3 Zhejiang University – Wanbangde pharmaceutical Group Joint Research Center for Chinese Medicine Modernization, Zhejiang Hangzhou, China

**Keywords:** *Ginkgo Biloba* Dropping Pill, Hypoxia/reoxygenation, Liver ischemia/reperfusion injury, AML-12 cells, Apoptosis, Inflammation

## Abstract

**Background:**

Liver ischemia/reperfusion (I/R) injury is an inevitable pathological phenomenon in various clinical conditions, such as liver transplantation, resection surgery, or shock, which is the major cause of morbidity and mortality after operation. *Ginkgo Biloba* Dropping Pill (GBDP) is a unique Chinese *Ginkgo Biloba* leaf extract preparation that exhibits a variety of beneficial biological activities. The aim of this study is to investigate the protective effects of GBDP on the liver I/R injury both in the in vitro and in vivo.

**Methods:**

Hypoxia/reoxygenation (H/R) experiments were performed in alpha mouse liver 12 (AML-12) cells and primary hepatocytes, which were pretreated with GBDP (60 or 120 µg/mL) followed by incubation in a hypoxia chamber. Cell viability was detected by 3-(4,5-dimethylthiazol-2-yl)-2.5-diphenyltetrazolium bromide (MTT) assay. Annexin V staining as well as western blot analysis of apoptosis-related proteins was performed to detect the protective effect of GBDP on cell apoptosis induced by H/R injury. C57BL/6 mice were used to establish the liver I/R injury model, and were pretreated with GBDP (100 or 200 mg/kg/day, i.g.) for two weeks. The liver damage was evaluated by detection of plasma levels of alanine transaminase (ALT) and aspartate transaminase (AST), as well as histopathological examinations. Liver inflammation was determined by detecting the secretion of pro-inflammatory cytokines and neutrophil infiltration through enzyme-linked immunosorbent assay (ELISA) and myeloperoxidase (MPO) immunohistochemistry staining. Finally, Terminal deoxynucleotidyl transferase-mediated dUTP-biotin nick and labeling (TUNEL) staining and western blot analysis of apoptosis-related proteins were used to investigate the anti-apoptotic effect of GBDP in mice.

**Results:**

In the in vitro study, GBDP pretreatment improved the cell viability of AML-12 cells in the H/R injury model. Similarly, the same result was found in the primary hepatocytes isolated from C57BL/6 mice. Moreover, GBDP decreased the number of apoptotic cells and reduced the expression of apoptosis-related proteins induced by H/R injury. In the in vivo study, oral administration of GBDP ameliorated liver injury evidenced by a significant decline in the levels of ALT and AST. Furthermore, the result of hematoxylin and eosin (H&E) staining showed that GBDP reduced the size of necrosis area in the liver tissue. In addition, the decreased infiltration of neutrophils and secretion of pro-inflammatory cytokines indicated that GBDP may play an anti-inflammatory effect. More importantly, GBDP reduced TUNEL-positive cells and the expression of apoptosis-related proteins in the liver indicating GBDP has anti-apoptotic effects.

**Conclusions:**

Our findings elucidated that GBDP has potential effects for protecting against liver I/R injury characterized by its anti-apoptotic, anti-necrotic, and anti-inflammatory properties, which would promisingly make contributions to the exploration of therapeutic strategies in the liver I/R injury.

## Background

Liver ischemia/reperfusion (I/R) injury is an inevitable pathological phenomenon in liver transplantation or liver resection, which is the major cause of morbidity and mortality after clinical liver transplantation [1]. Liver I/R injury leads to approximately 10% of early transplant failure and can result in a higher incidence of graft rejection [2]. Therefore, prevention and treatment of liver I/R injury should be addressed urgently in clinic. Despite extensive research, clinically effective interventions are still to be developed.

Liver I/R injury involves a biphasic process of ischemia-induced cell damage and reperfusion-induced inflammatory response [3]. When blood flow is interrupted, the cellular metabolism changes from aerobic to anaerobic due to the lack of oxygen supply, which can lead to various hepatocytes dysfunction [4]. Once the blood flows, various reactive oxygen species (ROS) are generated due to the reoxygenation of the ischemic liver tissue, which further aggravates the hepatocytes injury [5]. Liver I/R injury is charactered by hepatocyte damage, endothelial and kupffer cell swelling, neutrophil infiltration, vasoconstriction, ROS production, and platelet aggregation in sinusoids [1, 6]. Experimental evidence suggests that hepatocyte damage generally accompanies with hepatocellular necrosis and apoptosis, which are the major causes of hepatocytes death during liver I/R injury [7, 8]. As important regulators of the intrinsic apoptosis, B cell lymphoma-2 associated X (Bax), B cell lymphoma-2 (Bcl-2), poly ADP-ribose polymerase-1 (PARP-1), and cysteinyl aspartate specific proteinase (caspase) are involved in the initiation and execution of apoptosis, which is the key effect mechanism of liver I/R injury [9, 10]. In addition, inflammatory response is also a pathological mechanism of liver I/R injury. Kupffer cells are responsible for generation of pro-inflammatory factors, such as ROS and inflammatory cytokines to attract and activate neutrophils [4, 11, 12]. The infiltrated neutrophils further release myeloperoxidase (MPO), and pro-inflammatory cytokines and chemokines including interleukin (IL)-6 and monocyte chemoattractant protein (MCP)-1, all of which aggravate hepatocellular damage [1, 13]. Therefore, liver I/R injury is a series of events leading to necrosis, apoptosis, and hepatocytes dysfunction.

*Ginkgo biloba* L. is a traditional Chinese medicine commonly used to treat memory loss and improve blood circulation for thousands of years [14]. Recently, *Ginkgo biloba* leaf extract (GBE) is one of the most extensively used herbal medicine in the world [15]. It is reported that GBE has a variety of beneficial biological activities, including antioxidation, anti-inflammation, anti-tumor, cardioprotective and neuroprotective effects [16–20]. A standardized *Ginkgo biloba* leaf extract 761 (EGb 761) has been used as a strong antioxidative and neuroprotective agent to treat neurodegenerative diseases [21]. Furthermore, several researches have indicated that EGb 761 protects against renal, myocardial, and cerebral I/R injury [22–25], and improves hepatic DNA damage and liver microcirculation after warm ischemia [26, 27]. *Ginkgo biloba* Dropping Pill (GBDP) is a unique GBE preparation produced in China for treating angina pectoris and cerebral infarction caused by blood stasis. Each GBDP consists of 16 mg of GBE and 44 mg of polyethylene glycol 4000. A previous study reported that GBDP is different from EGb 761 in the content of components, and the quantitative analysis of 21 different components that identified between EGb 761 and GBDP indicated that EGb 761 has more organic acids compared to GBDP, whereas GBDP has higher levels of flavonols [28]. It has been reported that GBDP has antioxidative and neuroprotective effects in various conditions [29, 30]. Beyond that, the significant antagonistic effect of GBE on platelet-activating factor (PAF) receptor indicated that it may have beneficial effects on I/R injury. However, the effects of GBDP on liver I/R injury are still unclear. In this study, we investigated the effects of GBDP on hepatic I/R injury. The results showed that GBDP administration significantly inhibited liver injury by exhibiting anti-apoptotic, anti-necrotic, and anti-inflammatory effects both in the in vitro and in vivo. Taken together, as one of the most widely used Chinese medicine developed by modern science and technology worldwide, GBDP has a potential protective effect against liver I/R injury. Therefore, our study has a great significance in the application and development of GBDP, and provides a valuable strategy for the treatment of liver I/R injury.

## Materials and methods

### Reagents

GBDP (batch number: A01J180506) were provided by Wanbangde Pharmaceutical Group Co., Ltd (Wenling, Zhejiang, China). Dimethylsulfoxide (DMSO) and carboxymethyl cellulose sodium (CMC-Na) were purchased from Sinopharm Chemical Reagent Co., Ltd (Shanghai, China). Primary antibodies of Bax (2772S), caspase-3 (9662S), and β-actin (4970S) were purchased from Cell Signaling Technology (Beverly, MA, USA), Bcl-2 (ab196495) and PARP-1 (ab191217) were purchased from Abcam (Cambridge, MA, USA). Horseradish peroxidase (HRP)-conjugated secondary antibodies were purchased from Beyotime Biotechnology Co., Ltd (Shanghai, China). Enzyme-linked immunosorbent assay (ELISA) kits of MCP-1 and IL-6 were purchased from Invitrogen (Carlsbad, CA, USA).

### Animals

 Eight-week-old male C57BL/6 mice weighing 22–25 g were purchased from Beijing Vital River Laboratory Animal Technology Co., Ltd (Beijing, China) and maintained in a controlled environment (20–26 °C, 12 h light/dark cycle) with *ad libitum* access to food and water. The animal experiments were approved by the Animal Care and Use Committee of Zhejiang University School of Medicine.

### Isolation of primary hepatocytes and cell culture

Primary hepatocytes were isolated from C57BL/6 mice according to the method reported previously [4]. Briefly, primary hepatocytes were obtained by perfusion with 0.05% collagenase type IV purchased from Sigma-Aldrich (St. Louis, MO, USA). Then hepatocytes were filtrated through a 70 µm cell strainer and resuspended in media mixed with 42% percoll (Sigma-Aldrich, St. Louis, MO, USA) followed by centrifugation for 5 min at 1300 rpm. Hepatocytes were plated in the six-well culture dish at a density of 10^6^ cells/well and cultured in the Medium 199 (Sigma-Aldrich, St. Louis, MO, USA) supplemented with 10% fetal bovine serum (FBS) (Gibco, Invitrogen, Carlsbad, CA, USA), 1% penicillin-streptomycin (Gibco, Invitrogen, Carlsbad, CA, USA), 23 mM N-2-Hydroxyethylpiperazine-N-2-Ethane Sulfonic Acid (HEPES) (Sigma-Aldrich, St. Louis, MO, USA), and 10 nM dexamethasone (Sigma-Aldrich, St. Louis, MO, USA). The alpha mouse liver-12 (AML-12) cell line was purchased from the Type Culture Collection of the Chinese Academy of Sciences (Shanghai, China) and maintained in the Dulbecco’s modified Eagle’s medium (DMEM)/F12 (Gibco, Invitrogen, Carlsbad, CA, USA) containing 10% FBS, 1% penicillin-streptomycin, 1% Insulin-Transferrin-Selenium-G Supplement (ITS) (Sigma-Aldrich, St. Louis, MO, USA), and 40 ng/mL dexamethasone. Both AML-12 cells and primary hepatocytes in flasks were cultured in the cell incubator (Thermo Fisher Scientific, Waltham, MA, USA) at 37 °C with 5% CO_2_.

### Model of hypoxia/reoxygenation (H/R) injury

AML-12 cells or primary hepatocytes were incubated at 37 °C in a closed hypoxia chamber filled with N_2_ in a tri-gas incubator (94% N_2_, 5% CO_2_, 1% O_2_), followed by reoxygenation in normal culture conditions to establish the H/R injury model. Drug intervention with GBDP of two concentrations (60 and 120 µg/mL) was performed 1 h before the onset of hypoxia. The blank solution DMSO was served as control.

### Model of liver I/R injury and drug treatment

The mice were divided into four groups at random: sham, I/R, I/R + GBDP (100 mg/kg), I/R + GBDP (200 mg/kg). Mice in the I/R and I/R + GBDP groups went through 70% warm hepatic I/R injury as described previously [31]. Briefly, the mice were placed supine for midline laparotomy after anesthetization to expose the liver. The murine hepatic artery and portal vein were isolated and clipped with the microvascular clamp. After 45 min of ischemia, the clamp was removed. The samples of blood or liver tissue were collected after 6 or 24 h of reperfusion for subsequent experiments. The blood samples were centrifuged for 10 min at 4000 rpm, and the plasma was obtained for liver damage assessment. Mice in low- and high- dose I/R + GBDP groups were treated with GBDP in 1% CMC-Na by gavage once per day for two weeks, and 2 h before surgery on the 15th day. The 1% CMC-Na solution was given to the other two groups served as control.

### Cell viability assay

Cell viability was detected by 3-(4,5-dimethylthiazol-2-yl)-2.5-diphenyltetrazolium bromide (MTT) assay as described [32]. AML-12 cells or primary hepatocytes were seeded into a 96-well plate at a density of 1 × 10^4^ or 8 × 10^4^ cells/well respectively. Cells were cultured in H/R conditions after 1 h pretreatment of GBDP in different concentrations for 12 or 6 h. Then, the medium containing 0.5% MTT reagent was added to the each well. After incubation for 4 h, the supernatants were removed, and 100 µL DMSO was added to dissolve the formed formazan crystals at room temperature. The absorbance of the solution was measured at 580 nm using the Infinite M1000 Pro (TECAN, Mannedorf, Zurich, Switzerland).

### Annexin V staining

AML-12 cells were cultured in the hypoxia chamber for 12 h, and then reoxygenated for 22 h. Cells were stained with an Annexin V-fluorescein isothiocyanate (FITC)/propidium iodide (PI) apoptosis detection kit according to the manufacturer’s instructions (BD Biosciences, San Jose, CA, USA). Briefly, AML-12 cells seeded into 6-well plates were collected and resuspended in 1 × binding buffer to the final concentration of 1 × 10^6^ cells/mL. Then 100 µL cell resuspension was incubated with 5 µL Annexin V-FITC and 5 µL PI for 15 min at room temperature in the dark. Next, 400 µL 1 × binding buffer was added to end the incubation. Apoptotic rate was assayed by Accuri™ C6 flow cytometer (BD Bioscience, San Jose, CA, USA) in 1 h.

### Cell lysis and Western blot analysis

Preparation of whole cell or tissue lysates and western blot analysis were performed as described previously [33]. Briefly, the cells or tissues were lysed in the lysis buffer, which was made from Tris-HCL, NaCl, Ethylene Diamine Tetraacetic Acid (EDTA), glycerol, Triton X-100, Nonidet P-40, dithiothreitol, and phenylmethylsulfonyl fluoride, supplemented with protease and phosphatase inhibitors (Roche Diagnostics, Switzerland). The lysates were centrifuged for 15 min at 10,000 g, and the protein concentrations were quantified by Bradford method using Quick Start™ Bradford 1 × Dye Reagent (Bio-Rad, Hercules, CA, USA). Lysates with equal amount of six mice from the same group were mixed together to be one sample. Protein samples were electrophoresed by 9–12% sodium dodecyl sulphate–polyacrylamide gel electrophoresis (SDS-PAGE) gels (10–20 µg of protein/lane), and separated protein was transferred to polyvinylidene difluoride (PVDF) membranes (Merck Millipore, Darmstadt, Germany). The membranes were blocked in Tris-Buffered Saline with Tween 20 (TBST) containing 5% skim milk for 1 h at room temperature, which was followed by incubation with primary antibodies overnight at 4 °C. After washing three times in TBST, the membranes were incubated with the corresponding HRP-conjugated secondary antibodies for 1 h at room temperature. All primary antibodies were used at a 1:1000 dilution, and secondary antibodies were used at the dilution of 1:2000. Protein bands were visualized by using a ChemiDocTM XRS + system (Bio-Rad, Hercules, CA, USA) with chemiluminescence substrate reagents (Bio-Rad, Hercules, CA, USA) and quantified by using ImageJ software.

### Liver damage assessment

Plasma concentrations of alanine aminotransferase (ALT) and aspartate aminotransferase (AST), indicators of hepatocellular injury, were assayed by an automated biochemical analyzer (Cobas C8000, Roche Diagnostics, Switzerland) following the manufacturer’s instructions.

### Histological analysis

After fixation in 10% formaldehyde, the liver tissues were embedded in paraffin and sectioned at 5 µm thickness for hematoxylin and eosin (H&E), MPO immunohistochemical, and Terminal deoxynucleotidyl transferase-mediated dUTP-biotin nick and labeling (TUNEL) staining. The liver sections were stained with H&E to visualize the pattern in necrotic areas of the liver. The infiltration of neutrophils was detected by MPO staining. The liver sections were incubated with MPO primary antibody (1:1000, Servicebio, Wuhan, Hubei, China) at 37℃ for 1 h, followed by incubation with HRP-conjugated goat anti-mouse secondary antibody (1:200, Servicebio, Wuhan, Hubei, China). TUNEL staining was performed to determine DNA fragmentation using an In Situ Cell Death Detection Kit (Roche Diagnostics, Switzerland) according to the manufacturer’s instructions as described previously [34]. Images were captured using a fluorescence microscope (Nikon Eclipse Ti-SR, Tokyo, Japan).

### Statistical analysis

All statistical analyses were performed with GraphPad Prism 5.0, and the data was expressed as the mean ± standard deviation (SD). The statistical significance of the differences between two groups were calculated by two-tailed Student’s *t*-test, and one-way ANOVA with the Turkey’s post hoc test was used for comparisons among multiple groups. The *p* values less than 0.05 were considered statistically significant.

## Results

### GBDP alleviated the cellular injury of hepatocytes induced by H/R

**Fig. 1:**
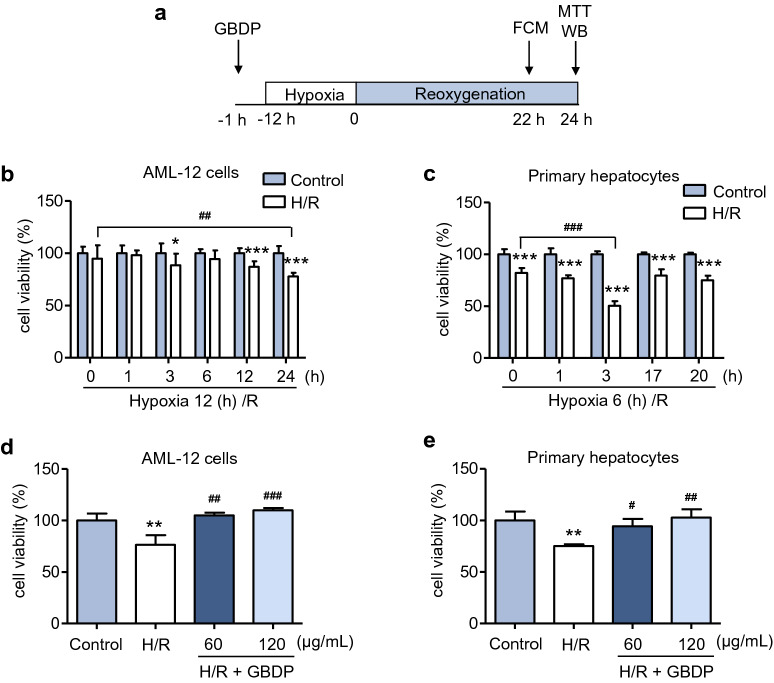
GBDP pretreatment improved the cell viability injured by H/R. **a** Flow diagram of the in vitro experiments. **b** Construction of hepatocellular H/R injury model. AML-12 cells or **c** primary hepatocytes were cultured in the hypoxia chamber for 12 or 6 h and reoxygenated for various time periods. The cell viability was determined by MTT assay. **d** AML-12 cells treated with or without GBDP (60 or 120 µg/mL) were cultured in the hypoxia chamber for 12 h and reoxygenated for 24 h. Cell viability was determined (n = 3 per group). **e** Primary hepatocytes were exposed to 6 h hypoxia and 3 h reoxygenation after pretreatment of GBDP, and cell viability was detected (n = 3 per group). *p < 0.05, **p < 0.01, and ***p < 0.001 vs Control group; ^#^*p* < 0.05, ^##^*p* < 0.01, and ^###^*p* < 0.001 vs H/R group

H/R, a commonly used model of ischemia/reperfusion injury in vitro, was performed to explore the extent of cellular damage [4]. AML-12 cells and primary hepatocytes were used to investigate the protective effects of GBDP against H/R-induced hepatocellular injury. Flow diagram of *in vitro* experiments was shown as Fig. 1a. We first investigated the time point of optimal H/R injury in AML-12 cells and primary hepatocytes. Results showed that the cell injury of AML-12 cells induced by H/R was maximized after 24 h of reperfusion (Fig. 1b), and the cell viability of primary hepatocytes fell to the lowest level after 3 h of reperfusion (Fig. 1c). Next, we constructed the H/R injury model of AML-12 cells or primary hepatocytes at indicated time point. Pretreatment of GBDP (60 or 120 µg/mL) improved the cell viability of AML-12 cells injured by H/R (Fig. 1d). Similarly, the same result can be found in primary hepatocytes (Fig. 1e). Altogether, these results indicated that GBDP has protective effects in hepatocellular injury induced by H/R.

### GBDP pretreatment inhibited apoptosis of hepatocytes induced by H/R

**Fig. 2:**
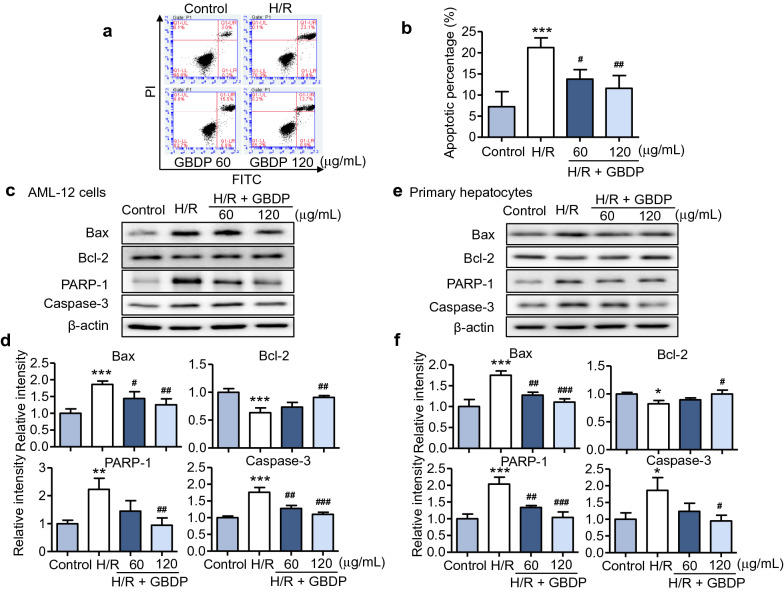
Apoptosis of hepatocytes induced by H/R injury was inhibited by GBDP pretreatment. **a** Apoptotic cells were determined by Annexin V staining in the AML-12 cells. Cells were pretreated with GBDP (60 or 120 µg/mL) for 1 h followed by incubation in the hypoxia chamber for 12 h and reoxygenation for 22 h. **b** Apoptotic rate of AML-12 cells was shown on the right (n = 3 per group). The protein expression of apoptosis-related markers was detected by western blot analysis. **c** AML-12 cells were pretreated with GBDP (60 or 120 µg/mL) for 1 h. The protein samples were collected after 12 h hypoxia and 24 h reoxygenation. The expression of Bax, Bcl-2, PARP-1, and caspase-3 was detected. **d** The quantification results were shown as below (n = 3 per group). **e** Primary hepatocytes were isolated from at least 3 different C57BL/6 mice, and pretreated with GBDP for 1 h. After 6 h hypoxia and 3 h reoxygenation, protein samples were obtained. The protein expression of apoptosis-related markers was determined.** f** The quantification results were shown as below (n = 3 per group). *p < 0.05, **p < 0.01, and ***p < 0.001 vs Control group; ^#^*p* < 0.05, ^##^*p* < 0.01, and ^###^*p* < 0.001 vs H/R group

Apoptosis is an important indicator, which reflects the degree of liver injury. The experiment of Annexin V staining was performed to determine the effect of GBDP on the apoptosis of AML-12 cells induced by H/R injury. As shown in Fig. 2a, H/R injury dramatically increased the number of annexin V-positive cells, while GBDP pretreatment dose-dependently decreased the apoptosis of AML-12 cells. The experiment was repeated at least three times and quantitative result of the apoptotic percentage was shown in Fig. 2b. Furthermore, the expression of apoptosis-related protein markers including Bax, Bcl-2, PARP-1, and caspase-3 was investigated by western blot analysis. GBDP pretreatment inhibited the expression changes of apoptosis-related proteins in AML-12 cells induced by H/R injury (Fig. 2c, d). Apart from the cell line, the expression of apoptosis-related proteins in primary hepatocytes has also been analyzed. Consistent with AML-12 cells, similar results were observed in primary hepatocytes (Fig. 2e, f). Overall, these results indicated that GBDP has a protective effect against H/R-induced apoptosis in hepatocytes.

### Oral administration of GBDP alleviated hepatic I/R injury in mice

**Fig. 3:**
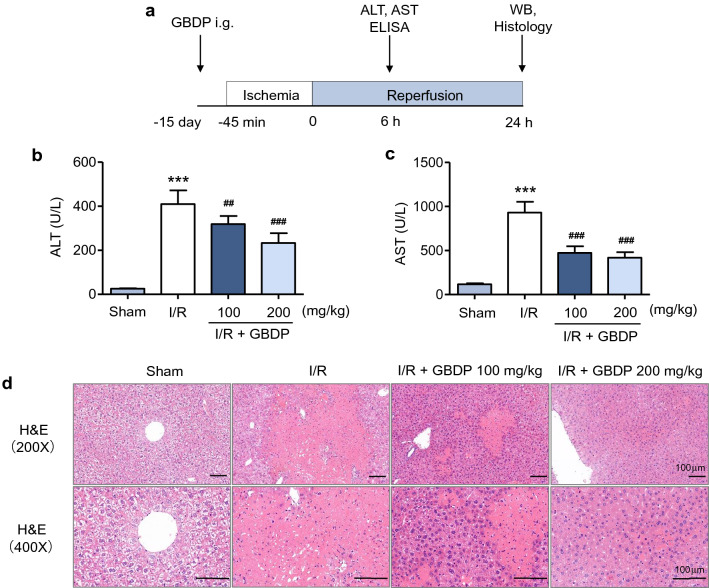
GBDP pretreatment attenuated the liver I/R injury in mice. **a** Flow diagram of the in vivo experiments. The samples of blood and liver tissue were collected, and the following analyses were performed: ALT, AST, and ELISA assay (6 h of reperfusion), western blotting for apoptotic proteins and histology (24 h of reperfusion). **b** The plasma levels of ALT and **c** AST were analyzed after 6 h of reperfusion. Values were expressed as mean ± standard error of the mean (n = 6 per group). **d** After 24 h of reperfusion, liver necrosis was assessed by H&E staining. Bars=100 μm.***p<0.001 vs Sham group;^##^*p*<0.01 and ^###^*p*<0.001 vs I/R group

To explore the in vivo efficacy of GBDP, we constructed a model of liver I/R injury in mice. Flow diagram of in vivo experiments was shown in the Fig. 3a. Mice were treated with GBDP by gavage once per day for two weeks, and 2 h prior to ischemia on the 15th day. The plasma levels of ALT and AST are sensitive indicators of acute liver injury, which go down after 24 h [35]. Thus, the blood samples were collected after 6 h of reperfusion. The levels of ALT and AST were significantly elevated after liver I/R injury, while GBDP administration decreased them in a dose-dependent manner (Fig. 3b, c). More importantly, the result of H&E staining showed that both low and high doses of GBDP significantly reduced the liver necrosis, which was found widely in the I/R group (Fig. 3d). These findings indicated that GBDP plays a potential role to protect against liver I/R injury in mice.

### GBDP inhibited liver inflammation induced by I/R injury in mice

**Fig. 4:**
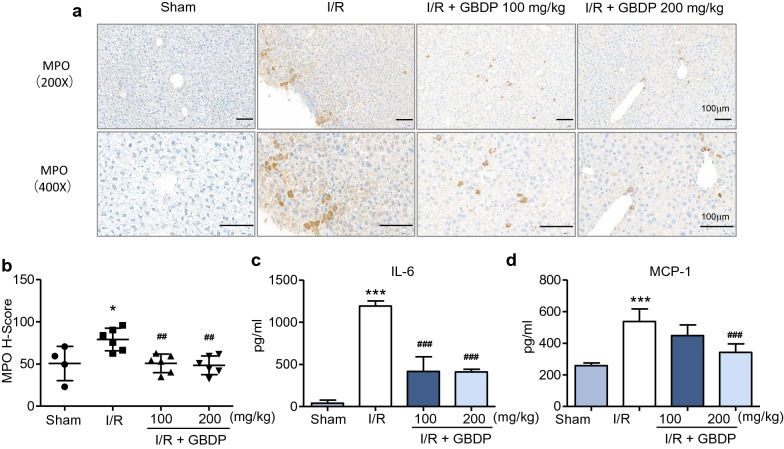
GBDP administration attenuated the inflammatory response in I/R-injured liver. **a** After 24 h of reperfusion, neutrophil infiltration was assessed by MPO immunohistochemical staining. Bars = 100 μm. **b** Histochemistry score of MPO was shown (n = 4-6 per group). **c** The plasma levels of IL-6 and **d** MCP-1 were analyzed by ELISA kits (n = 6 per group). *p<0.05 and ***p<0.001 vs Sham group; ^##^*p*<0.01 and ^###^*p*<0.001 vs I/R group

To further verify the protective role of GBDP on the liver inflammation after I/R injury, MPO immunohistochemical staining and ELISA assay were performed to detect the neutrophil infiltration and secretion of pro-inflammatory cytokines respectively. MPO is an enzyme predominantly stored in neutrophil granules, which can be used to quantify neutrophil infiltration in the liver [4]. The results showed that the number of MPO-positive cells was significantly reduced by GBDP administration, indicating that the hepatic infiltration of neutrophils was inhibited (Fig. 4a). Meanwhile, MPO H-Score also declined in GBDP-treated groups (Fig. 4b). Besides, the decreased plasma levels of pro-inflammatory cytokine (IL-6) and chemokine (MCP-1) were also observed in GBDP-treated groups in Fig. 4c, d. These results further confirmed the inhibitory effects of GBDP on inflammatory response caused by I/R injury.

### GBDP attenuated hepatocytes apoptosis induced by liver I/R injury in mice

**Fig. 5:**
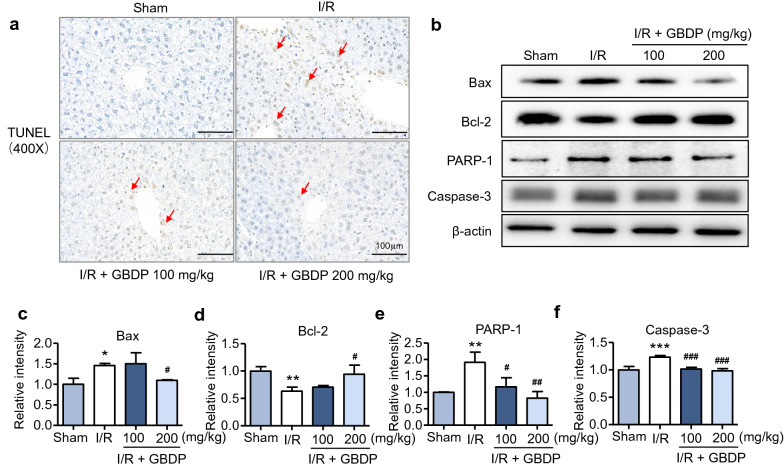
Liver I/R injury-induced hepatocellular apoptosis was inhibited by GBDP pretreatment in mice. **a** Apoptotic cells were detected by TUNEL staining after 24 h of reperfusion. Bars = 100 μm. **b** The protein expression of apoptosis-related markers (Bax, Bcl-2, PARP-1, and caspase-3) in reperfused liver tissue was analyzed by western blotting. The lysate sample of each group was a mixture of 6 mice from the same group (n = 6 per group). β-actin was used as a loading control. The mixture sample of each group was prepared 3 times and the experiments were repeated at least 3 times. **c** The quantification results of Bax, **d** Bcl-2, **e** PARP-1, and **f **caspase-3 were shown as below. *p<0.05, **p<0.01, and ***p<0.001 vs Sham group;^#^*p*<0.05, ^##^*p*<0.01, and ^###^*p*<0.001 vs I/R group

**Fig. 6:**
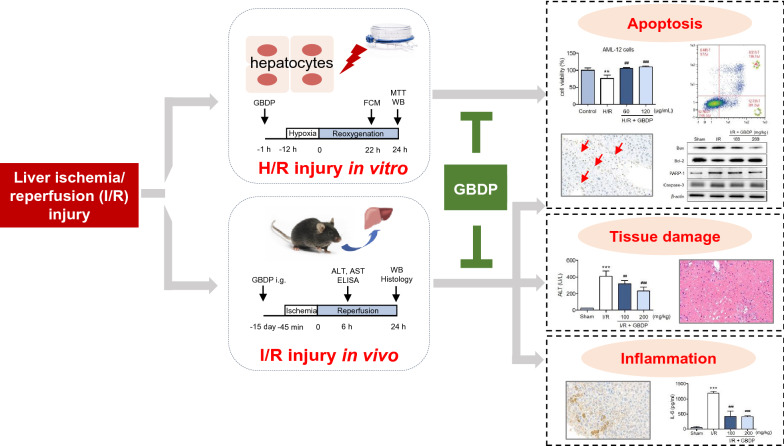
A brief diagram of this study was shown. GBDP significantly inhibited H/R-induced hepatocytes injury in vitro and I/R-induced liver injury in vivo

In addition to liver necrosis and inflammation, hepatocytes apoptosis induced by liver I/R injury has also been explored. Firstly, the number of apoptotic cells in the liver tissue was determined by TUNEL staining. Both low and high doses of GBDP massively decreased the number of apoptotic cells, which was markedly increased in the I/R group (Fig. 5a). Next, the expression of apoptosis-related proteins including Bax, Bcl-2, PARP-1, and caspase-3 in the liver tissue were detected by western blot analysis. The mixed lysate sample of each group was prepared as described previously [34]. Consistent with the histological results, GBDP downregulated the expression of pro-apoptotic proteins (Bax, PARP-1, and caspase-3) elevated by I/R injury (Fig. 5b). Meanwhile, GBDP up-regulated the expression of anti-apoptotic protein (Bcl-2) reduced by I/R injury in Fig. 5b. The experiments were performed at least three times, and quantitative results of Bax, Bcl-2, PARP-1, and caspase-3 were shown in Fig. 5c–f. These results demonstrated that GBDP has a strong inhibitory effect on liver I/R injury-induced hepatocellular apoptosis in mice. Altogether, GBDP displays protective effects against liver I/R injury by suppressing apoptosis, liver necrosis, and inflammation. A brief diagram of this study was shown in Fig. 6.

## Discussion

Liver transplantation is an effective therapeutic method for treatment of end-stage liver disease. However, there is a huge disparity between the number of liver organs available for transplantation and the number of patients waiting for the liver transplantation, leading to an increase in the mortality of patients on the waiting list [6]. Therefore, it is important to improve the success rate of liver transplantation for a small number of patients who have received the donor liver. Liver I/R injury is the major underlying cause of graft non-function or late dysfunction after liver transplantation [36]. Hence, prevention and reduction of I/R injury are the key factors for successful liver transplantation.

As one of the most universally used herbal medicinal products [37], GBE has a wide range of pharmacological properties. It is well known that the liver I/R injury generally accompanies with apoptosis, inflammation, and ROS generation [3]. Numerous studies have shown that GBE has the characters of inflammation reduction, free radical scavenging, anti-tumor and nervous system activity enhancement [19, 38, 39]. GBDP is a unique and popular GBE preparation in China produced according to the Pharmacopoeia of the People’s Republic of China (Edition 2015). The main active components of GBDP including terpene trilactones, flavonoids, bioflavonoids, and organic acids. The flavonoids in GBDP are mainly divided into flavonoids and their glycosides, diflavonoids, and catechins. The terpene trilactones are the unique components of *Ginkgo biloba* L., containing diterpenoids (Ginkgolide A, B, C, etc.) and sesquiterpenes (bilobalide). Terpene trilactones and flavonoids are the most important active components in GBDP. It has been reported that flavonoids have strong anti-inflammatory, antioxidative, and anti-apoptotic effects, which may have potential protective effects against liver I/R injury [40–42]. Moreover, as the unique active components of GBDP, ginkgolides and bilobalide have been found to exhibit an antagonistic effect on PAF receptor specifically, which is deemed to associate with the protective effect in I/R injury [43–45]. For this condition, we hypothesized GBDP could be an alternative therapy for preventing liver I/R injury.

In this study, we first conducted hepatocytes H/R injury model in vitro, a commonly used tool in liver I/R research to mimic cellular damage in the pathological process of I/R injury[4]. GBDP pretreatment significantly increased the cell viability of AML-12 cells after H/R stimulation. Primary hepatocytes provide an acceptable reflection of the hepatic in vivo situation [46], and the same result observed in primary hepatocytes further confirmed our conclusion that GBDP protected against cellular injury induced by H/R stimulation.

Apoptosis of hepatocytes is one of the most important types of cell death in the progression of hepatic I/R injury [47]. Hepatocyte dies through active suicide in response to the overwhelming cellular stress, which is termed “apoptosis” [48]. During this physiological process, the activation of caspases results in the cleavage of proteins, then activation of nucleases that cleave DNA into fragments subsequently, leading to cell death [49]. Cells with fragmented DNA could be identified as apoptotic cells by TUNEL assay, which is a common detection way of apoptosis [50]. Furthermore, apoptosis is controlled by multiple genes including Bax, Bcl-2, PARP-1, and caspase family. Overexpression of Bax, a pro-apoptotic protein, could cause caspase-9 mediated programmed cell death and ultimately upregulate caspase-3, which is considered as a final step towards apoptosis [51]. In our results, flow cytometry data showed that the apoptosis induced by H/R injury was dose-dependently alleviated by GBDP pretreatment in AML-12 cells. Meanwhile, the expression changes of apoptosis-related protein markers induced by H/R injury were also suppressed by GBDP both in the AML-12 cells and primary hepatocytes. Consistent with the results of the in vitro experiments, subsequent animal experiments confirmed that GBDP administration reduced hepatocytes apoptosis caused by liver I/R injury, which has been shown in downregulating the expression of pro-apoptotic proteins and reducing the number of TUNEL-positive cells. Similar to these results, GBE has been reported to reduce the protein levels of Bax and caspase-3 in the liver fibrosis model, which further confirmed the hepatoprotective effect of GBE through the anti-apoptotic effect [52].

Sterile inflammation is one of the characteristics of liver I/R injury, which is marked by abundant infiltration of neutrophils and secretion of pro-inflammatory factors [53]. Intravital microscopy was used in mice hepatic I/R injury model to show massive recruitment of neutrophils to the site of hepatic injury [54]. Accumulated neutrophils in the hepatic parenchyma release ROS, proteases, and inflammatory factors, which results in hepatocytes damage and hepatic sinusoids destruction [55]. Thereby, several studies emphasized the strategies of inhibiting neutrophils recruitment to reduce I/R-induced liver injury [56, 57]. Analysis of infiltrated neutrophils in the liver has been mainly assessed by histologic sections or activated markers such as MPO [54]. MPO is a peroxidase enzyme and most abundantly expressed in neutrophils. It is stored in azurophilic granules of the neutrophils and release into the extracellular space upon neutrophils recruitment [4]. In some ways, the level of MPO represents the degree of neutrophils infiltration. Therefore, we performed the immunohistochemical staining of MPO to analyze the infiltration of neutrophils in our study. Consistent with the anti-inflammatory effect of GBE [58], GBDP downregulated the infiltration of neutrophils in the liver as well as the secretion levels of IL-6 and MCP-1. Further experiments should be performed to study the protective mechanisms of GBDP on liver I/R injury by interfering with inflammatory responses.

## Conclusions

In conclusion, the present study elucidated that GBDP exerted a protective function on liver I/R injury both in the in vitro and in vivo, as indicated by reducing apoptosis, liver necrosis, and inflammatory response. Overall, our results demonstrated a beneficial function of GBDP that may be expected to be a candidate therapeutic agent to prevent liver I/R injury.

## Data Availability

All data and materials in the current study are included in this published article.

## Abbreviations

ALT: Alanine aminotransferase
AML-12: Alpha mouse liver-12
AST: Aspartate aminotransferase
Bax: B cell lymphoma-2 associated X
Bcl-2: B cell lymphoma-2
Caspase: Cysteinyl aspartate specific proteinase
CMC-Na: Carboxymethyl cellulose sodium
DMEM: Dulbecco's modified Eagle's medium
DMSO: Dimethyl sulfoxide
EDTA: Ethylene Diamine Tetraacetic Acid
ELISA: Enzyme-linked immunosorbent assay
FBS: Fetal bovine serum
FITC: Fluorescein isothiocyanate
GBDP: *Ginkgo Biloba *Dropping Pill
GBE: *Ginkgo Biloba* leaf extract
H&E: Hematoxylin and eosin
HEPES: N-2-Hydroxyethylpiperazine-N-2-Ethane Sulfonic Acid
H/R: Hypoxia/reoxygenation
HRP: Horseradish peroxidase
I/R: Ischemia/reperfusion
IL-6: Interleukin-6
ITS: Insulin-Transferrin-Sielenium-G Supplement
MCP-1: Monocyte chemoattractant protein-1
MPO: Myeloperoxidase
MTT: 3-(4,5-dimethylthiazol-2-yl)-2.5-diphenyltetrazolium bromide
PAF: Platelet-activating factor
PARP-1: Poly ADP-ribose polymerase-1
SDS-PAGE: Sodium dodecyl sulphate–polyacrylamide gel electrophoresis
PI: Propidium iodide
PVDF: Polyvinylidene difluoride
ROS: Reactive oxygen species
SD: Standard deviation
TBST: Tris-buffered saline with tween 20
TUNEL: Terminal deoxynucleotidyl transferase-mediated dUTP-biotin nick and labeling
